# Toward a cognitive-developmental understanding of mental toughness

**DOI:** 10.3389/fpsyg.2026.1843811

**Published:** 2026-07-14

**Authors:** Merle Berg, Konstantinos Velentzas, Thomas Schack

**Affiliations:** 1 Department of Sport Science, University Bielefeld, Bielefeld, Germany; 2 Cognitive Interaction Technology – Center of Excellence CITEC, University Bielefeld, Bielefeld, Germany

**Keywords:** athlete development, mental toughness, performance enhancement, psychological skills, regulatory strategies

## Abstract

**Theoretical background:**

Mental toughness (*MT*) is a crucial psychological construct that enables athletes to perform high-level sports. Despite extensive research, MT suffers from conceptual ambiguity that oscillates between trait- and state-based interpretations. Despite this controversy, a widely accepted developmental framework of mental toughness, known as the Mental Toughness Pyramid has received widespread acceptance in the scientific field of sport psychology, delivering its fundamentals to researchers and practitioners.

**Aim:**

The aim of this study is to critically reflect on existing research, theoretical models, and approaches regarding the concept of MT to provide new theoretical evidence that will help extend and specify the current understanding and work in the specific field. Specifically, our attempt is to enhance Mental Toughness Pyramid model precision by adding a cognitive-affective perspective and experience level. This approach can facilitate the work of researchers and practitioners.

**Methods:**

A semantic analysis and review of existing literature with the help of a database search was conducted primarily using Google Scholar and PubMed to identify relevant studies on the topic of MT and applied sport psychology. The initial focus was on review articles and foundational literature, which served as the basis for expanding the search to additional relevant sources. This approach ensured a thorough understanding of the current state of research and key theoretical frameworks in the field. In order to elaborate on existing findings, a link to further domains (e.g., psychological skills training, routine, and self-regulation) was established.

**Conclusion:**

The debate now shifts from a trait vs. state dichotomy to understanding MT as a multifaceted construct shaped by dynamic interactions among environment, personality, cognition, behavior, learning, and experience. From this point of view, we suggest an extension of Mental Toughness Pyramid in order to enhance the precision of researchers’ and practitioners’ work.

## Introduction

Mental toughness (*MT*) is regarded as a key psychological model that refers to psychological skills that enable individuals to thrive in demanding competitive sporting situations (Gucciardi et al., 2015). Accordingly, Gerber (2011) concludes that MT is highly correlated to flow experience as well as stress management, emotional regulation, performance motivation, and volition and is related to stable and enhanced performance. This perspective is also supported by several theoretical assumptions and empirical evidence, as demonstrated by the review conducted by Guszkowska and Wójcik (2021), which concluded that MT leads to better athletic performance across various sports. For example, volleyball players with higher MT scores show significantly higher serve effectiveness (Mack, 2019), wrestlers win more fights (Drees and Mack, 2012), and handball players exhibit increased goal effectiveness with high MT scores (Ragab, 2015). Therefore, it is not surprising that MT is categorized by Olympic athletes, coaches, and sports psychologists as the most important psychological construct in sports (Gerber, 2011; Gould et al., 2002). Despite its acknowledged importance, MT remains a model with “poor conceptual clarity” (Gucciardi, 2017, p.18), as numerous definitions and models continue to coexist. This conceptual ambiguity extends to questions on development and trainability. Accordingly, the aim of this study is to contribute to greater conceptual clarity in order to facilitate the work of researchers and practitioners regarding MT. To this end, key stages in the development of the construct are outlined and critically examined, with particular attention given to identifying existing limitations and inconsistencies within the literature. Building on this analysis, a global and more integrative perspective is adopted in order to address and potentially resolve these shortcomings. From this perspective, the scientific community will be able to analyze the relevant components in more detail and gain an understanding of MT development. The consequence of such procedures is the optimization of practitioners’ work. Sport psychological interventions can become more effective so that athletes can more quickly acquire psychoregulatory skills, stabilizing and/or enhancing performance.

## Methods

To review and critically discuss the current understanding of MT, the foundational literature was consulted as an initial step. In particular, the body of work conducted by the research group around Jones et al. (2002, 2007) and Connaughton et al. (2010) was identified as especially influential. To better understand the development of MT, the study and developmental model proposed by Bull et al. (2005) were considered. Building on this, an extensive literature search was conducted. Relevant literature was first identified through an exploratory search using Google Scholar to obtain an overview of the research field. This was followed by a more systematic literature search in PubMed using specific search terms and filters (e.g., “mental toughness,” “performance”). The review process began with key review articles (e.g., Cowden, 2017; Crust, 2008; Guszkowska and Wójcik, 2021; Liew et al., 2019) to establish an overview of the current understanding of MT and its relationship to performance. Subsequently, the literature was further divided into distinct research directions, such as the conceptualization of MT as a state or trait (e.g., Cattell et al., 1955; Gucciardi et al., 2015) and the trainability of MT (e.g., Beattie et al., 2019; Crust and Azadi, 2010; Golby and Sheard, 2006; Ragab, 2015). To provide a comprehensive framework for situating MT, related and embedded theoretical domains were also included. These comprised concepts from cognitive behavioral theory (Ellis, 1955, 1991; Beck, 1963, 2015), self-efficacy (Bandura, 1977, 1986), and routines (Velentzas et al., 2010; Velentzas et al., 2011; Lidor, 2007; Lidor et al., 2014).

### Theoretical foundations of mental toughness

#### Definition

Commonalities in definitions of MT indicate that it implies all relevant psychological skills that are essential in order to cope with challenging or stressful situations. This means that MT allows athletes to remain resilient and/or regain control of their performance, showing similar effects to the use of coping strategies (Crust and Azadi, 2010; Guszkowska and Wójcik, 2021; Liew et al., 2019). However, the MT measurement criteria regarding several attempts to elaborate on their effects are inconsistent. From this perspective, earlier conceptualizations often used opponents’ performance as a comparative basis, whereas more recent definitions emphasize subjective and goal-directed dimensions (Gucciardi, 2017). Following up, the author states that the content of the definitions varies from specific behaviors to fundamental characteristics. Cattell et al. (1955)—already in the early 1960s—also highlighted MT’s influence on success in sport. Additionally, Cattell (1957) described ‘tough-mindedness’ as a *personality trait* that should represent the ability to separate oneself from the pressure, feelings, and problems of others. In essence, Cattell agreed that *tough-minded* individuals are more self-confident, realistic, responsible, and show low emotional sensitivity (see also Liew et al., 2019; Unkrig, 2022). Accordingly, Loehr (1982) described MT as the capacity to mobilize and direct energy in a constructive manner during crises, combined with the maintenance of a positive attitude toward challenging and demanding situations. However, Loehr’s work has been criticized for its lack of precision, scientific rigor, and rational conceptualization (Crust, 2008; Liew et al., 2019). Goldberg (2005) emphasized MT as the functional handling of failures closely related to resilience and perseverance. In contrast to Cattell (1957), both Loehr (1982) and Goldberg (2005) state that MT can be developed and learned (i.e., Liew et al., 2019; Loehr, 1994). Therefore, they assume that learning coping strategies and enhancing, for example, self-control and self-confidence, lead to the development of MT. Because these outlooks are based on anecdotes and observations, criticism arose regarding superficiality and insufficient scientific foundation (Unkrig, 2022). In an attempt to provide more scientific evidence regarding MT, Fourie and Potgieter (2001) conducted surveys with 131 elite coaches and 160 elite athletes, implementing one of the first empirical investigations into MT. After an inductive content analysis of all participant answers, the authors identified 12 characteristics, ranked by importance, defining mentally tough athletes (Table 1; Fourie and Potgieter, 2001). Athletes and coaches weighed these characteristics differently. The coaches emphasized concentration and focus, whereas the athletes considered perseverance and determination to be the most important characteristics.

**Table 1:** Twelve characteristics of MT by Fourie & Potgieter.

Characteristics	Themes
Motivational level	Perseverance, determination, desire, responsibility and commitment
Coping skills	Coping ability, composure, acceptance, activation control and adaptability
Confidence maintenance	Competence, self-confidence and attitude
Cognitive skill	Concentration ability, focus ability, thinking ability, decision-making ability and analysing ability
Discipline and goal-directedness	Discipline, goal-orientation and idealism
Competitiveness	Will, appearance of a winner, consistent performance, high competitive level and big match temperament
Possession of prerequisite physical and mental requirements	High physical and mental conditioning, ability to cope with pain and self-sacrifice
Team Unity	Ability to reveal respect, team cohesion and relationship skills
Preparation skills	Balance, balanced preparation and visualisation
Psychological hardiness	Strong personality, emotional and psychological well-being, charge-taking and autonomy
Religious convictions	Religious beliefs
Ethics	Sense of righteousness

The first definition that met scientific standards was published the following year.

MT is a natural or developed psychological edge that enables athletes to (1) generally cope better than their opponents with the many sports demands (competition, training, and lifestyle) and (2) specifically be more consistent and better than their opponents in staying determined, focused, confident, and in control under pressure (Jones et al., 2002, p. 209).

In line with Fourie and Potgieter (2001), Jones et al. (2002) identified 12 characteristics of MT that incorporated the perspectives of athletes, coaches, and sports psychologists (Table 2).

**Table 2:** Twelve characteristics of MT by Jones et al. (2002).

Rank	Mental toughness characteristic
1	having an unshakable self-belief in one’s ability to achieve your competition goals
2	bouncing back from performance setbacks as a result of increased determination to succeed
3	having an unshakable self-belief that one’s possess unique qualities and abilities
4	having an insatiable desire and internalised motives to succeed
5	remaining fully-focused on the task at hand in the face of competition-specific distractions
6	regaining psychological control following competition-specific unexpected and uncontrollable events
7	pushing back the boundaries of physical and emotional pain, while still maintaining technique and effort under distress (in training and competition)
8	accepting that competition anxiety is inevitable and knowing that one can cope with it
9	thriving on the pressure of competition
10	not being adversely affected by others’ good and bad performances
11	remaining fully-focused in the face of personal life distractions
12	switching a sport focus on and off as required

In addition, the conceptualization of MT proposed by Jones et al. (2002) was supported by subsequent studies, such as the research conducted by Thelwell et al. (2005), which followed the recommendation to investigate MT within a single-sport context. In this context, they conducted semi-structured interviews asking six international soccer players’ opinions about the definition and attributes of MT. A total of 10 characteristics were identified that showed a high degree of agreement with those proposed by Jones et al. (2002). These attributes were further evaluated and ranked in terms of importance by 43 professional male soccer players in a subsequent study (Thelwell et al., 2005). Based on these findings, the conceptualization proposed by Jones et al. (2002) is considered highly credible (Gerber, 2011). Further exploration of MT using *Personal Construct Theory* (Kelly, 1955) was conducted by Jones et al. (2007). Following this, eight elite athletes (Olympic or World Champions) were asked about MT, which confirmed the previously stated definition (Crust, 2008) and extended MT-related characteristics. Consequently, 30 attributes were identified, which were subsequently sorted into four dimensions, forming one of the most popular MT models. The first dimension comprises *attitude and mindset*, which include *belief* in oneself and *focus*. The second dimension deals with the implementation of regulatory strategies in the *training* environment, including the use of goals and the ability to go beyond one’s own limits. The third dimension includes behavioral and cognitive regulatory strategies in a *competitive* environment. The fourth and last dimension involves dealing with *post-competition* failure and success and covering regulatory competences after performance. However, criticism arises through several methodological aspects, such as the verification of a threshold between mentally tough and mentally weak athletes and/or a focus on an athlete’s given performance (e.g., elite-performing athletes). From this perspective, the main question arises regarding several confounding variables that could shape MT (Gerber, 2011; Jones et al., 2007).

Overall, both the definition and conceptualization proposed by Jones et al. (2002, 2007) have received substantial support from other research groups (e.g., Bull et al., 2005; Thelwell et al., 2005). Nevertheless, some criticisms persist. These include reliance on the assumption that successful athletes inherently exhibit MT, inclusion of performance outcomes in the definition, small sample sizes, and a lack of theoretical grounding (Crust, 2008; Gerber, 2011). Furthermore, there is a lack of reference to existing theoretical constructs, making it difficult to differentiate between them.

Other attempts to clarify the concept of MT include related constructs such as hardiness and resilience. For instance, Clough et al. (2002) proposed the 4C model of MT, which is based on the concept of hardiness—a personality trait comprising three dimensions: control, challenge, and commitment (Kobasa, 1979). They further incorporated confidence as a fourth dimension to define MT (Gerber, 2011; Gucciardi, 2017; Unkrig, 2022). However, the model is criticized for its lack of theoretical distinction from hardiness, which blurs the conceptual boundaries between performance and health psychology while having only a marginal relation to performance and skill enhancement (Gucciardi, 2017).

Similar to the definition proposed by Goldberg (2005), many researchers attempting to clarify the concept of MT have drawn parallels between MT and resilience (Unkrig, 2022). This is defined as “the process by which one bounces back or recovers from such major assaults […]” (Gucciardi et al., 2015, p. 29) and “the capacity of a dynamic system to adapt successfully to disturbances that threaten its function, viability, or development” (Gucciardi, 2017, p. 19). Consequently, Gucciardi (2017) suggests that resilience and MT share the characteristic of adapting to stressful and pressurized situations. However, resilience extends beyond the individual to groups or systems and involves multiple protective factors, including biological, social, societal, and psychological dimensions. MT can be assigned to the psychological dimension, which can therefore be defined as a subcategory of resilience. Finally, resilience is a reaction to stressors, while MT is purposeful and therefore includes both proactive (e.g., competition planning) and reactive (e.g., dealing with injuries) action tendencies. Hence, MT represents a protective factor in the course of resilience but should not be conflated with it (Gucciardi, 2017; Gucciardi et al., 2015; Mendizabal, 2024).

Traditionally, MT has been associated with factors such as *goal orientation*, *determination*, *control beliefs*, *self-confidence*, the *ability to thrive under pressure*, and the *use of regulatory strategies* (Fourie and Potgieter, 2001; Jones et al., 2002). This conceptualization primarily emphasizes the positive outcomes, characteristics, and behaviors associated with MT and defines related action tendencies. However, this approach has been criticized by Gucciardi (2017) because of the exclusion of “other potentially relevant qualities that reflect those features that tie them together” (p.18). The author offers an alternative definition, describing MT as a “state-like, psychological resource that is purposeful, flexible, and efficient in nature for the enactment and maintenance of goal-directed pursuits” (Gucciardi, 2017, p.18). Similarly, Cowden (2017) conceptualizes MT as a psychological differentiator that “facilitates the consistent pursuit of performance excellence” (p.2). The above-mentioned definitions reflect attempts to identify a common denominator for the diverse characteristics and behaviors associated with MT, highlighting unresolved questions regarding both the nature of MT and its malleability.

Although Loehr (1982) proposed that MT can be developed early on, much of the literature—predominantly conducted with elite athletes—has implicitly adopted a dichotomous perspective, portraying athletes as either mentally tough and therefore successful or lacking MT and consequently underperforming. While the behavioral attributes of MT are now well documented, it remains unclear whether MT should be understood merely as a cluster of psychological attributes or as a broader, dynamic construct, as suggested by Gucciardi (2017).

Likewise, the debate on the conceptual status of MT has evolved. Initially framed as a relatively stable personality trait (e.g., Cattell, 1957), more recent perspectives emphasize state-like and developmental qualities (e.g., Gucciardi, 2017). Furthermore, although a positive association between MT and performance is widely assumed (Singh, 2014), the direction of causality remains unresolved. MT may facilitate superior performance; however, repeated successes may also strengthen MT.

#### Development

**Figure 1:**
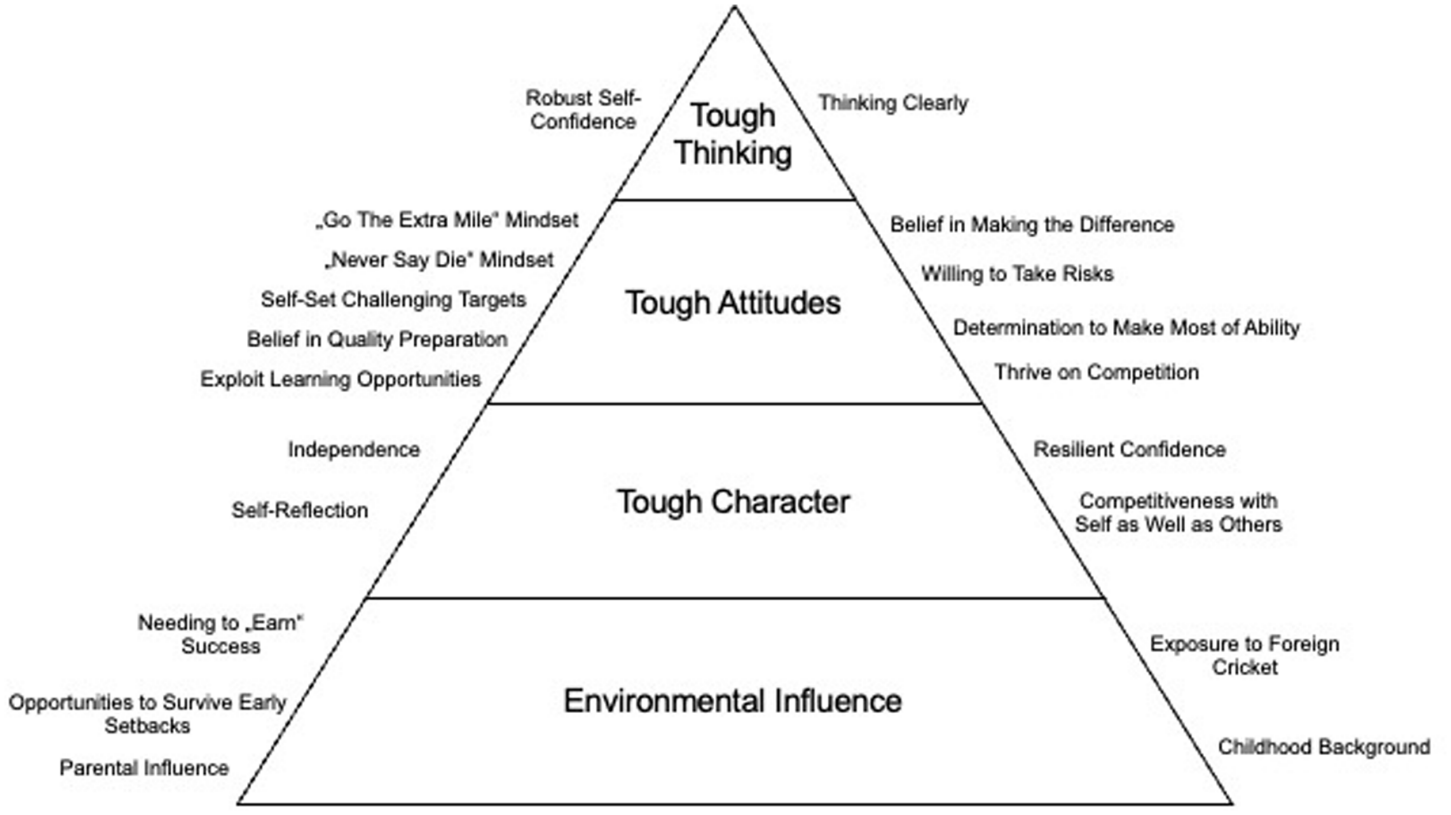
Mental Toughness Pyramid adapted from Bull et al. (2005).

When considering MT and the state–trait discussion, scientific research into the development of MT characteristics is indispensable. Two development models have emerged in this context, with athletic performance as a mutual criterion. The first is the development pyramid proposed by Bull et al. (2005), known as the Mental Toughness Pyramid (Figure 1). The model is based on focused interviews with elite cricket players. Initially, 101 cricket coaches were surveyed about the mentally toughest players during the 1980s and 1990s. During the selection process, the research group decided to use cultural consensus analysis to establish a common frame for MT instead of predefined concepts from sport psychology. A total of 12 coach-nominated players were chosen for in-depth interviews regarding their *winning minds*, *MT*, *MT development*, *effects on pressure situations*, *personal signs of MT,* and *tips for young athletes seeking to develop MT*. A special feature of the study was the existing co-working background between the interviewer and participants. This was leveraged by adopting an informal approach during the 40- to 80-min interviews. Through semantic analysis, 733 quotes were identified and summarized into five comprehensive dimensions, which showed similarities to Jones et al.’s (2002) definition: (1) *Development Factors*, (2) *Personal Responsibility*, (3) *Dedication and Commitment*, (4) *Belief,* and (5) *Coping with Pressure*. From these dimensions, four hierarchical levels of the development pyramid (*Environmental Influence, Tough Character, Tough Attitudes, and Tough Thinking*) were derived. Initially, environmental influences were emphasized as the foundation for the development of MT. They are divided into two primary strands: upbringing and transition into an appropriate cricket environment. Factors such as *parental influence* and *opportunities to survive early setbacks* have been cited in this context. The next level encompasses *Tough Character*, representing personality variables and associated traits. These traits include *independence*, *sense of responsibility, self-reflection, competitiveness,* and *confidence*. The authors highlight the importance of these stable traits for the effectiveness (e.g., stability) of the attitudes and thinking patterns exhibited by mentally tough athletes. The subsequent level addresses *Tough Attitudes*, referring to behavioral patterns applied to specific situations and acquired based on the *Tough Character*. These patterns include the use of *goal setting*, *thriving on competition,* and *willingness to take risks*. At the top of the pyramid are thought patterns that are influenced by an individual’s ability to concentrate, psychological control, and acceptance of competitive anxiety. From the practitioners’ point of view, most sport psychology interventions are primarily targeted at this top level (Bull et al., 2005). The authors emphasize the significant influence of environmental factors, which in turn affect character development. These two emphasized aspects expand the existing understanding of MT, which has traditionally focused on the higher levels of the pyramid, pointing only to the top of the MT iceberg. Furthermore, the authors highlight that the connection between *Tough Characters* and *Tough Attitudes* is crucial for the development of a winning mindset (Bull et al., 2005). Accordingly, Gerber (2011) concludes that the combination of these two levels represents the fundamental components of MT. Although attitudes and cognitive patterns can be developed, their full effectiveness depends on the presence of the corresponding character traits (Bull et al., 2005). Despite emphasizing traits in their model, the authors propose a long-term analytical approach, in which athletic performance and/or sport psychology interventions should be considered. Under these circumstances, the authors implicitly incorporate the state perspective within their pyramid (Bull et al., 2005). Their recommendation is in line with past investigations, which assumed that MT is significantly related to performance. This performance-based perspective is plausible from a scientific standpoint but simultaneously leads to the improbable assumption that high-performance athletes must share the same personality traits or a similar upbringing. Furthermore, it remains unclear whether recreational and/or intermediate athletes also possess or develop MT.

Support for Bull et al.’s (2005) MT developmental framework can be found in several subsequent studies investigating the origins of MT. In particular, environmental influences appear to play a central role in the emergence and development of mentally challenging behaviors. For example, Crust and Clough (2011) emphasized the importance of challenging yet supportive learning environments that encourage independent problem-solving, personal responsibility, gradual exposure to demanding situations, and reflective processing of failure experiences. Similarly, Anthony et al. (2016), synthesizing findings on MT development, highlighted the importance of supportive social environments, including relationships with coaches, parents, and teammates, as central developmental resources. These findings align closely with previous observations emphasizing coach–athlete relationships and supportive practice environments as important contributors to MT development (Butt et al., 2010; Connaughton et al., 2008; Gucciardi et al., 2009). In addition to the importance of environmental influences, the authors suggest that MT development involves dynamic cognitive-affective and self-regulatory characteristics. The conceptualization of MT is related to personal characteristics as malleable psychological resources that can be developed and adapted across different contexts through learning and experience. This assumption integrates past scientific results, including those of Mahoney et al. (2014), who highlighted the importance of heightened awareness; Driska et al. (2012), who emphasized the significance of cognitive strategies; and Connaughton et al. (2010), who integrated the importance of reflective practice in the MT development process.

**Figure 2:**
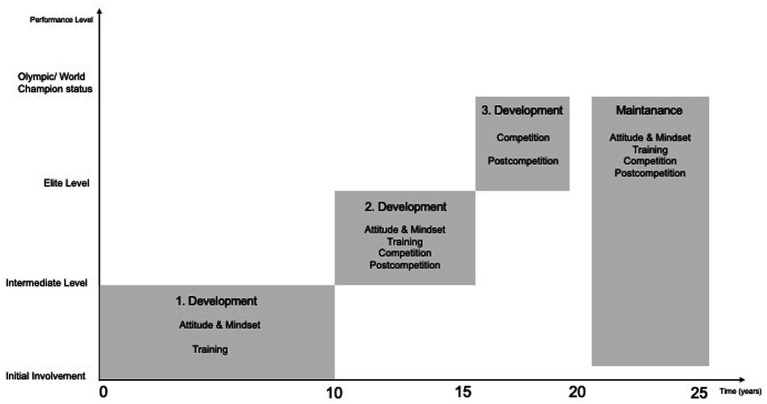
Developmental model of mental toughness adapted from Connaughton et al. (2010).

Whereas Bull et al.’s (2005) pyramid attempts to clarify the fundamentals of MT development, Connaughton et al. (2010) introduced a new model that was based on Jones et al.’s (2007) framework. The authors used a time and performance axis in the model by outlining a developmental trajectory of MT that emerges progressively from an initially non-existent state across three stages (early, middle, and later years) and a maintenance phase. This progression is accompanied by a corresponding increase in performance levels (initial involvement to the intermediate level, intermediate to elite level, and elite level to Olympic/world champion status). The authors argue that the development of MT is shaped by several underlying mechanisms, including *effective leadership, exposure to elite performers, supportive social networks,* and *accumulated competitive experience*. Across all three developmental phases, both positive and adverse life events were considered potential contributors to growth. Furthermore, the systematic acquisition of mental skills and regulatory strategies is advocated, proposing that MT is best conceptualized as a state-like construct that fluctuates over time and needs to be actively maintained (Connaughton et al., 2010) (see Figure 2).

Connaughton et al. (2010) based the development of MT in athletes on the acquisition and application of regulatory strategies. According to their model, the longer and more intensive the engagement with a specific task or skill—under professional monitoring—the greater the number of regulatory strategies learned and the higher the probability that an athlete will demonstrate MT. The authors assume that the acquisition of regulatory strategies represents the most essential procedure of MT. From this point of view, the developmental model pays less attention to the influence of traits than the development pyramid (Bull et al., 2005). Consequently, the authors focus on higher levels of psychological ability. Accordingly, it has been suggested that they promote MT as a state-like construct that can be learned and acquired.

The similarities between the models can be mainly attributed to the upper pyramid level (e.g., *Tough Thinking*). Additionally, they show the influences of environmental factors, such as opportunities for modeling, support from parents and coaches, rivalry, and the rationalization of success and failure. However, a comparison of the two models suggests that their outcomes remain contradictory. On the one hand, the MT pyramid (Bull et al., 2005) highlights the importance of an athlete’s personality (trait) in the development of *Tough Thinking*. On the other hand, Connaughton et al. (2010) emphasize that the regular use and application of self-regulatory strategies (state) are fundamental to building MT. However, it remains unclear to what extent the application of regulatory strategies constitutes a sufficient criterion for athletes’ MT. At the same time, it is ambiguous whether performance significantly influences MT development or whether MT development influences performance.

### Empirical evidence on state, trait, and trainability perspectives of mental toughness

Despite MT’s acknowledged importance, the coexistence of numerous definitions and models enhances the necessity of more conceptual clarity (Gucciardi, 2017). This conceptual ambiguity extends to questions on development and trainability. In particular, the debate on whether MT should be understood primarily as a stable trait (Cattell, 1957) or as a fluctuating state (Jones et al., 2007; Connaughton et al., 2010) remains unresolved.

The state–trait discussion is rooted in Spielberger’s theory, which distinguishes between situational, fluctuating states, and stable traits, defined as a person’s predisposition to react consistently to the same stimulus with certain emotions or behaviors (Spielberger, 1966; Spielberger et al., 1971). According to this assumption, researchers in the early years suggested that MT could be understood as a personality trait. Therefore, Cattell (1957) was among the first researchers to address MT from a trait perspective by incorporating *tough-mindedness* into sensitivity, which is one of the 16 primary personality traits (Crust, 2008).

Another way to examine MT as a personality trait is to compare it to existing personality theories such as the *Big Five personality factors* (McCrae and Costa, 1997). Using a combination method that included both twin and adoption data, researchers confirmed the genetic influence of the *Big Five factors*. However, when attributing the influence of genetics and environment to personality, research has demonstrated a margin of error of approximately 20% (Oerter and Montada, 2008). Oshio et al. (2018) used this approach to clarify the meaning of resilience. Resilience essentially encompasses both states and traits (Child and Medvedev, 2024), although Oshio et al. (2018) refers exclusively to ego-resilience and trait resilience (i.e., only the trait components). The results showed significant correlations with the *Big Five*. Extending this approach, Horsburgh et al. (2009) analyzed a sample of 219 monozygotic and dizygotic twins to investigate their associations with *Big Five* personality traits. Using the 48-item Mental Toughness Questionnaire (Clough et al., 2002), the authors found significant correlations between MT and four of the five personality factors (*openness, conscientiousness, extraversion, and agreeableness*). Therefore, their results indicate a hereditary component of the sub-dimensions and an overall MT index of 36 and 56%, respectively. Accordingly, the authors suggest that this corresponds to a heritability percentage that can be used as an indicator for a personality trait. Further, the authors attribute the interindividual differences to environmental influences, leaving open the possibility that MT could also be related to psychological states (Horsburgh et al., 2009). Supporting the trait perspective, Hardy et al. (2014) chose another analytical approach using the Mental Toughness Inventory (MTI). During peer rating, participants were asked to complete the questionnaire for a peer athlete (third-person rating). With 59 sports science students assessing informant-rated mental behavior, the study demonstrated almost excellent test–retest reliability (*r = 0.96*). The results describe the stability of MT over 3 weeks.

However, the test–retest methodology and data collection often used to analyze MT have been heavily criticized (Gucciardi et al., 2015). The authors pointed out that participants were mostly asked about their general and typical thoughts, emotions, and behavior, stating that this approach influences the results toward the trait assumption. At the same time, they assume that the focus on high performance also restricts insights into MT’s nature. Therefore, in their study (Gucciardi et al., 2015), they considered state characteristics during MT analysis, offering more details. They conducted a *weekly review* of 203 undergraduate sport science students from an Australian university over 10 weeks. Participants were asked to complete the Mental Toughness Index (Gucciardi et al., 2015), which contains questions about *MT*, including information about *goal progress*, *thriving,* and *psychological health*. They attributed 44% of the total variance in MT to interpersonal differences, and 56% to intraindividual differences. In line with Gucciardi’s research group (2017), several studies, conducted over longer periods, show variation that questions the previously assumed trait approach and brings the state position up for discussion (e.g., Bell et al., 2013; Gucciardi, 2017; Sheard and Golby, 2006). This assumption confirms and complements previous statements, such as those of Jones et al. (2007), who suggested that the MT of high-performance athletes can both develop and fluctuate during their careers. To investigate the maintenance of MT, 10 elite athletes from the original study by Jones et al. (2002) were re-interviewed (Connaughton et al., 2008). By applying semi-structured interviews, the authors identified the peak of MT after 3 years of competition experience at the top level, thereby highlighting MT’s temporal development and malleability. The authors attribute essential roles to internalized motivation to succeed, the support network, and the use of psychological strategies in the long-term development and maintenance of MT.

During the MT state position becomes more prominent; several researchers have paid attention to the bidirectional influence on MT and interventions. Following this tendency, Sheard and Golby (2006) conducted a seven-week psychological skills training program with 36 national-level swimmers, including *goal setting, visualization, relaxation techniques,* and *thought control*. In a pre-, post-, and retention design, with individual regulatory strategy sessions (45 min each), the authors analyzed changes in MT. The characteristics were measured via self-reports, which depicted the perception of *success, MT, hardiness, self-esteem, self-efficacy, and optimism.* Performance changes in different strokes, measured by performance times, were also considered. The results showed significant enhancements in 17 of the 18 positive psychological measures and improved swimming performance.

Gucciardi et al. (2009) compared the differences between teaching regulatory strategies and specific MT training by conducting a six-week intervention study with Australian football players. The first intervention group received psychological skills training that focused on *self-regulation, arousal regulation, mental rehearsal, attentional control, self-efficacy,* and *ideal performance*. The second group received MT training, which included *personal and team values, work ethics, self-motivation, self-belief, concentration and focus, resilience, emotional intelligence, sport intelligence,* and *physical toughness*. The results were measured using ratings from parents, coaches, and athletes and reports on resilience and flow. Improvements were demonstrated in all measured dependent variables. In comparison to the control group, there were significant differences in various dimensions of *MT*, *resilience*, and *flow*. Minimal differences between intervention types are likely to reflect overlapping content. Therefore, the researchers conclude that MT can be developed through interventions focusing on the development of psychological skills.

Crust and Azadi (2010) substantiated the previous approach to influencing MT by examining the relationship between MT and the use of regulatory strategies. For this purpose, 67 men and 40 women acting on different performance levels (club/university to national level) completed the Mental Toughness Questionnaire-48 (MTQ48; Clough et al., 2002) and the Test of Performance Strategies (TOPS; Thomas et al., 1999). The results showed significant correlations between *self-talk, emotional control, relaxation strategies,* and *MT. Commitment*—as a subscale of the MTQ48—correlated the most with the use of regulatory strategies (Crust and Azadi, 2010). The results supported the MT development thesis, highlighting the positive influence of regulatory strategies on MT and performance.

Bell et al. (2013) conducted a longitudinal MT intervention that specifically focused on improving performance under pressure. Over a two-year course program, 41 elite youth cricket players learned coping strategies for dealing with pressure in three classroom-based workshops on preparation principles and psychological skills, as well as through individual support from a sports psychologist. Punishments in this phase were used to create a pressurized situation. Repeated measures of the MTI (Hardy et al., 2014), competitive performance statistics, and an indoor fitness test showed significant improvements in coach-rated mentally tough behaviors, objectively assessed competitive performance statistics, indoor batting against pace, and a multistage fitness test compared to the control group. No differences were observed in batting against spin. Although the study did not explicitly focus on psychological skills training, the results showed a significant relationship between MT development and performance enhancement.

In contrast, Ragab (2015) focused on regulatory strategies and MT training but approached it from a reversed perspective, examining the effects of MT training on coping skills and throwing effectiveness in university handball players. Training was performed three times a week over a period of 8 weeks, focusing on five pillars: (a) Winning is not your sole responsibility; (b) You are not just a handball player; (c) You cannot control everything; (d) Staying positive is not enough; and (e) Stuff happens (see also Ragab, 2015, p.432). This study used the MTQ48 and Athletic Coping Skills Inventory (Smith et al., 1995) questionnaires and shooting tests. The results showed a significant increase in *MT, athletic coping skills,* and *shooting effectiveness* in the intervention group. The results indicated a bidirectional relationship between regulatory strategies and MT.

Another way to influence MT is through cognitive-behavioral approaches such as *Situational Instruction Training* Meichenbaum and Fitzpatrick (1993). This was implemented in the study by Slack et al. (2015), who proposed an alternative method for developing or maintaining MT. Based on cognitive-behavioral approaches such as Meichenbaum and Fitzpatrick (1993) *Situational Instruction Training,* the authors conducted the Mental Toughness Education and Training Programme (MTETP) in elite football officiating. The MT of the three English Football League referees was measured using the MTQ48 (Clough et al., 2002), Referee-Specific MT (Slack et al., 2013, 2014), and Football Association match-day referee-assessor reports. Data collection was conducted in the baseline phase and three times during the intervention. The results showed positive changes in the general and specific MT attributes (e.g., behavior and cognition). In the case of poor performance, a decline in MT cognition self-rating was observed. Therefore, the authors concluded that MT is both stable and dynamic in nature, indicating traits as well as the state nature of MT.

Similarly, Berg (2024) used a cognitive-behavioral approach, referring to the Rational-Emotive Therapy (RET) by Ellis (1955). The author conducted an intervention study with competitive youth handball players (intervention vs. control) based on the cognitive intervention approach (Craemer and Craemer, 2014), specifically following RET Ellis (1955), focusing on athletes’ irrational beliefs relativization. The Mental Toughness Inventory (Middleton et al., 2005) was used to measure MT. The results showed a significant improvement in 9 of the 13 MTI factors (e.g., *goal commitment, task focus,* and *self-concept*). Improvements in *self-efficacy, stress minimization, potential, perseverance, positive attitudes,* and *global MT* were highly significant. From this point of view, Berg (2024) assumed that MT can develop not only through regulatory strategy training and environmental factors but also through an optimization of the fundamental cognitive attributes.

### Critical view on current mental toughness research

Despite different methodological approaches and scientific goals, a common denominator is the mutual relationship between MT, regulatory strategies, and performance. Within the state–trait debate and despite the existing tendencies, it remains unclear how these two concepts interact to facilitate several stages of MT development. This lack of consensus may partly stem from the diverse theoretical and methodological approaches applied across studies, as well as from the predominant focus on high-performance athletes, which may limit the generalizability of existing frameworks. Conceptualizations of MT have predominantly used a top-down procedure to describe the manifestation and behavioral characteristics of high-performing athletes. While some authors emphasize the stability of the construct and argue for a predominantly trait-like interpretation (e.g., Cattell et al., 1955; Horsburgh et al., 2009), others highlight its situational variability (e.g., Gucciardi et al., 2015; Jones et al., 2007). Under these circumstances, Bull et al. (2005) attempted to integrate both perspectives in their model, simultaneously paying more attention to the traits while providing fewer explanations regarding the underlying cognitive-developmental mechanisms through which MT emerges and develops over time.

The evolution of perspectives on MT is clear: early research favored the trait perspective (e.g., Cattell et al., 1955; Cattell, 1957), demonstrating a connection to the *Big Five* and a heritability index of 56% in twin studies (Horsburgh et al., 2009) and test–retest stability over 3 weeks (Hardy et al., 2014). Jones et al. (2007) marked a turning point in the conceptualization of MT and its development. Based on Jones et al.’s (2007) framework, several studies have highlighted fluctuations, developmental changes, loss, and intrapersonal variability during athletes’ careers (Connaughton et al., 2010; Gucciardi, 2017; Jones et al., 2007). Furthermore, clear changes were observed during the long-term evaluation of MT development based on specific psychological skill training (e.g., psychological skills training, pressure management interventions, or cognitive-behavioral approaches). Consequently, the focus has shifted from trait to state views. Despite this, a growing consensus suggests that both nature and nurture coexist in this discussion, and both perspectives remain theoretically relevant (Gerber, 2011).

Even though Bull et al. (2005) research group separates trait and state pyramid levels (*Thought Character* implies traits, and *Thought Attitudes* and *Thinking* imply states), the authors themselves indicate substantial interrelations between these levels. This interdependence suggests that the lower levels of the pyramid may not merely represent stable dispositional foundations, but rather dynamic cognitive-affective structures interacting continuously with athletes’ behavior. From this perspective, current MT frameworks may overemphasize the regulation and optimization of mentally tough behavior (e.g., through routines, self-regulation strategies, and psychological skills training) while paying comparatively less attention to the cognitive structures and experiential processes that enable such behaviors to emerge naturally and adaptively under pressure.

An additional perspective may, therefore, be linked to the cognitive-behavioral outlook—most prominently advanced by Ellis (1955) and Beck (1963). Despite their differing theoretical emphases, both authors posit that human behavior—including sport performance—is fundamentally shaped by underlying cognitive appraisals and structured patterns of thinking (e.g., *Tough Thinking*). Ellis (1991) differentiates between rational and irrational beliefs, suggesting that maladaptive emotional and behavioral responses are largely rooted in rigid and illogical belief systems. In contrast, Beck (2015) highlights the role of negative automatic thoughts, cognitive distortions, and core beliefs as central mechanisms influencing affect and behavior. These cognitive structures are generally conceptualized as relatively stable and enduring over time. Given their structural stability and regulatory function, it appears theoretically justified to extend the construct of the *Tough Character* by incorporating these cognitive dimensions. Integrating these cognitive components may contribute to a more comprehensive and conceptually robust understanding of MT development.

Consequently, we assume that the state–trait debate may be insufficient to fully capture the complexity of MT, suggesting instead that MT should be understood as a dynamic and multifaceted construct. Following Jones et al.’s (2007) recommendation, future research should focus less on categorizing MT exclusively as either a state or trait and more on clarifying its developmental nature and underlying mechanisms. Future research may benefit from shifting attention to predominantly top-down regulatory approaches. Despite the demonstrated improvements in MT, this predominantly top-down approach appears theoretically inconsistent with the developmental assumptions proposed by Bull et al. (2005), who conceptualize MT development as a bottom-up process originating from environmental influences. This perspective aligns with the findings of Cowden (2017), who emphasized environmental factors as particularly influential in the development of MT.

Considering this, researchers must prioritize the clarification of the subcomponents included in MT from a semantic perspective (bottom-up analysis). In the second step, the research focus should be directed toward the weighting of these subcomponents in the measurement and development of MT. Whereas Cowden (2017) defines environmental factors as the most influential, other researchers focus on the upper levels of the pyramid (Connaughton et al., 2010).

Furthermore, existing research has predominantly focused on the upper levels of Bull et al.’s (2005) pyramid (*Tough Attitudes* and *Thinking*), aiming primarily to structure and optimize observable MT characteristics. In contrast, comparatively limited attention has been paid to the *Tough Character* level, despite its potential importance in understanding how mentally tough behaviors are cognitively and experientially developed over time.

*Tough Character* consequently determines whether environmental factors are utilized to shape subsequent developmental stages (traits and states). Such an approach can extend the reciprocal interaction to the upper levels of Bull et al.’s (2005) pyramid (Figure 1) because, from our perspective, *Tough Character* implies alterable states, such as hardiness, that influence performance under pressure, which can be reflected on the *Tough Attitudes* level, affecting willingness to take risks, setting challenging targets, or establishing the “never-say-die” mindset. Therefore, from our point of view, the process of understanding MT should be reversed, starting from the lower levels of Bull et al.’s (2005) pyramid, which means that the *Tough Character* level should be the focus of future research. The relevant states and their significant influence on the upper levels (e.g., *Tough Attitudes* and *Tough Thinking*) must be clarified. Accordingly, we expect that this approach may improve the theoretical understanding of MT development and support the refinement of future research strategies targeting both evaluation and development through MT intervention.

Our assumption is in line with Bandura (1986) social learning theory, in which environment, cognition, and personality shape human behavior. The author attributes differences in behavior among athletes to previous learning experiences rather than genetics and situational factors. Bandura (1997) viewed personality as the result of reciprocal interactions. According to this theory, personality is a variable that can adapt depending on learning experiences such as operant conditioning and modeling, as well as environmental influences (Bandura, 1986; Bandura, 1997). Also in line with this is Anthony et al. (2016) research, describing experiences as crucial components of MT development—especially when athletes operate in supportive environments and have sufficient psychological preparedness. Therefore, from our point of view, experience can be conceptualized not only as a participant characteristic but also as an integral component of the developmental pyramid proposed by Bull et al. (2005). Such integration will support a dynamic reciprocal feedback system between *Tough Character, Experience, and Tough Attitudes*, which in turn may continuously influence all other levels of the model.

### Extension of the mental toughness framework as a dynamic model

Building on the increasing empirical and theoretical support for Bull et al.’s (2005) developmental pyramid, we assume that the framework provides a valuable basis for understanding the developmental nature of MT. As outlined previously, several findings support the relevance of environmental influences, experiences, cognitive-affective processes, and self-regulatory mechanisms in MT development. However, to date, these findings have not been systematically integrated into a comprehensive model. Furthermore, some interactions within the framework remain underexplained or conceptually unspecific. Accordingly, the present extension focuses primarily on a further elaboration of the *Tough Character* level and the introduction of an additional *Experience* level to more explicitly capture the reciprocal developmental interactions underlying MT.

Following the developmental logic proposed by Bull et al. (2005), the present extension adopts a bottom-up theoretical and scientific perspective, beginning with environmental influences. As the relevance of environmental factors for MT development has already been widely supported in previous research, this level does not require substantial reconceptualization. However, environmental influences may continuously shape the *Tough Character* level through learning, modeling, exposure to adversity, and motivational climate. In this regard, indirect mechanisms such as passive gene–environment correlation (e.g., Plomin et al., 1977) may additionally contribute to the interaction between dispositional tendencies and environmental developmental conditions.

From our point of view, the *Tough Character* level can be understood as the central developmental interface that determines how environmental influences are perceived, utilized, and translated into developmental processes. Within the present extension, *Tough Character* is therefore not conceptualized as a purely stable dispositional foundation but rather as a combination of both relatively stable psychological structures and more dynamic characteristics and regulatory processes continuously interacting with environmental conditions and experiences.

Relatively stable psychological structures may include personality-related predispositions such as the *Big Five* characteristics, resilient confidence, independence, action orientation, hardiness, self-concept, core beliefs (Beck, 2015), and irrational beliefs (Ellis, 1991). Although these structures demonstrate stability over time, they remain modifiable through experience and continuous cognitive evaluation. In addition, the *Tough Character* level may include more dynamic regulatory processes such as attentional focus, emotional regulation, self-reflection, and situational confidence. These processes may continuously regulate how athletes interpret environmental demands, process experiences, and behaviorally respond to pressure situations.

**Figure 3:**
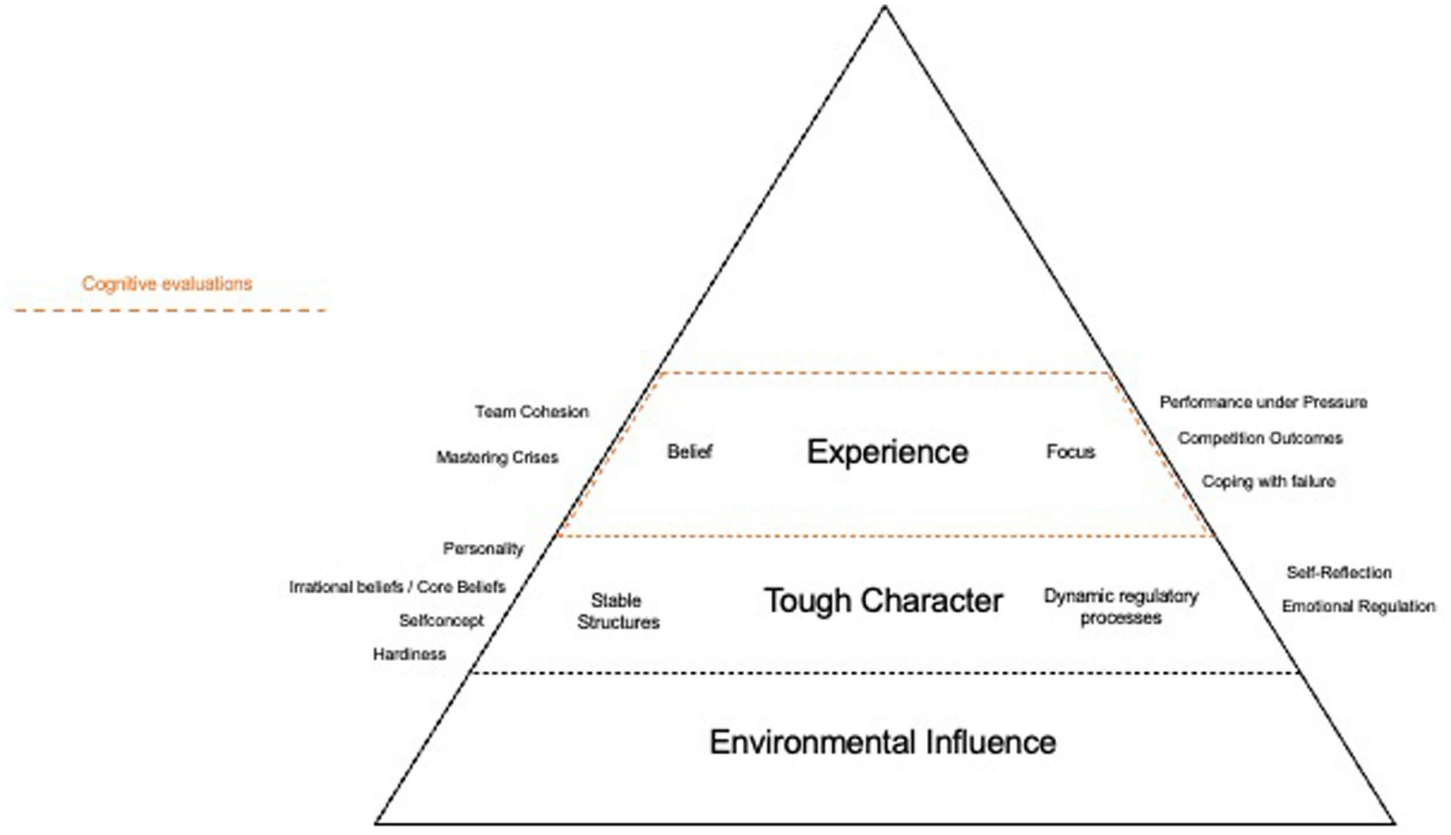
Proposed cognitive-developmental extension of the Mental Toughness Pyramid.

However, environmental influences and the *Tough Character* alone may not sufficiently explain the development of mentally tough behavior. Therefore, the present extension introduces *Experience* as an additional intermediary developmental dimension positioned between *Tough Character* and *Tough Attitudes*. From this perspective, experiences may represent the central developmental mechanism through which environmental conditions and psychological structures are translated into attitudes and thinking patterns. Experiences such as success, failure, injury, crises, team cohesion, and performance under pressure are not interpreted objectively, but rather cognitively evaluated through existing belief systems, self-concept, attentional focus, and appraisal processes. Consequently, identical experiences may influence MT development differently depending on the athlete’s underlying psychological structures and regulatory processes. For example, successful experiences may strengthen self-belief, resilient confidence, and perceived competence, whereas failure experiences may either facilitate adaptive focus, self-reflection, and coping processes or reinforce maladaptive beliefs and dysfunctional cognitive patterns. Accordingly, cognitive evaluation processes may determine whether experiences contribute to functional or dysfunctional psychological adaptations. At the same time, the resulting experiences and cognitive evaluations may continuously influence the underlying *Tough Character* structures. Repeated experiences may strengthen or weaken one’s self-concept, beliefs, attentional tendencies, and other psychological structures over time. Simultaneously, the resulting psychological characteristics may further influence how athletes perceive environmental conditions, seek developmental opportunities, and behaviorally engage in future experiences. Consequently, environmental influences through the *Tough Character* and *Experience* may operate as a continuously adaptive reciprocal feedback system (see Figure 3).

Within the present extension, the upper levels (*Tough Attitudes* and *Tough Thinking*) are conceptualized less as independent developmental origins and more as adaptive manifestations emerging from the reciprocal interaction between environmental influences, psychological structures, regulatory processes, and experiences. From this point of view, theoretical models and applied work should not focus only on these two upper levels to identify MT. Accordingly, attitudes such as determination, willingness to take risks, persistence, “never-say-die” mentality, and belief in preparation may develop through repeated adaptive interactions between experience and cognitive evaluation processes. Similarly, *Tough Thinking* may reflect the resulting adaptive thinking patterns, robust self-confidence, self-regulatory processes, and cognitive strategies developed throughout athletes’ careers. Importantly, these thinking patterns may subsequently influence future experiences and cognitive evaluations, thereby continuously affecting the underlying *Tough Character* structures and future developmental pathways.

**Figure 4:**
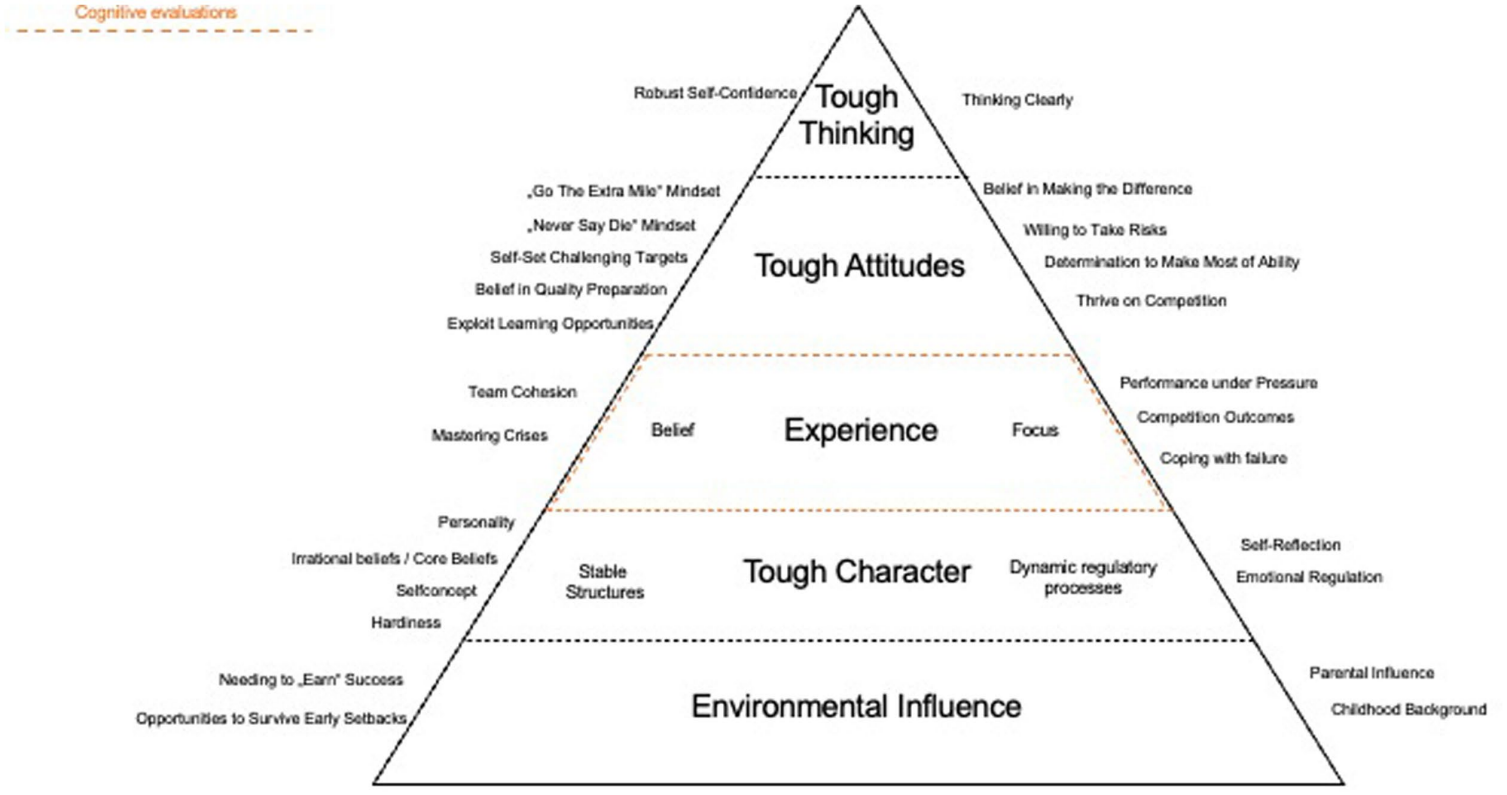
Extended Mental Toughness Pyramid illustrating a dynamic developmental model based on Bull et al.’s (2005) framework and the reciprocal interaction between environmental influences, Tough Character, experiences, Tough Attitudes, and Tough Thinking.

Consequently, *Experience* and mental states also appear to operate within a continuously adaptive relationship. This reciprocal perspective can provide a promising framework for future MT research by allowing researchers to investigate not only the behavioral manifestations of MT but also the cognitive-developmental processes underlying its emergence and adaptation over time. From this perspective, a more precise identification and weighting of relevant psychological states and cognitive-affective processes may be essential for advancing the conceptual understanding of MT and its development. Initial attempts in this direction were made by Berg (2024), who investigated irrational beliefs within MT-related cognitive processes, following Craemer and Craemer (2014) (see Figure 4).

Accordingly, in future research, it will be necessary to evaluate and weigh the traits, states, and cognitive components that are most important for MT development. Therefore, we suggest, through our extension, that scientists in the field of MT should emphasize identifying and specifying dynamically which states can be assigned to the respective levels of the pyramid. This approach contributes to both (a) theoretical precision and (b) improved practical applicability. This approach can be achieved through existing methods such as a *think-aloud protocol*, an extension of existing questionnaires, or even the development of a new diagnostic instrument (Farnsworth et al., 2021). Furthermore, future studies should investigate the relationship between performance development, experience, and MT in greater detail, particularly by including lower-performing athletic populations. Such an approach may help clarify the generalizability of existing MT models beyond elite sport contexts while providing further insight into the role of developmental experiences and performance progression in the emergence of MT. A more precise understanding of MT development may enable sport psychology practitioners to design more targeted and theoretically grounded interventions (e.g., psychological skills training) to enhance MT and ultimately improve athletic performance.
